# Elucidating the Sound Absorption Characteristics of Foxtail Millet (*Setariaitalica*) Husk

**DOI:** 10.3390/ma13225126

**Published:** 2020-11-13

**Authors:** Dhayalini Balasubramanian, Senthil Rajendran, Bhuvanesh Srinivasan, Nirmalakumari Angamuthu

**Affiliations:** 1 Structural Engineering Division, Department of Civil Engineering, Anna University, Chennai 600 025, Tamil Nadu, India; senthilr68@gmail.com; 2 CNRS-Saint Gobain-NIMS, UMI 3629, Laboratory for Innovative Key Materials and Structures (LINK), National Institute for Materials Science, Tsukuba 305-0044, Japan; SRINIVASAN.Bhuvanesh@nims.go.jp; 3 Center of Excellence in Millets, Tamil Nadu Agricultural University, Athiyandal, Tiruvannamalai 606 603, Tamil Nadu, India; anirmalakumari@yahoo.com

**Keywords:** foxtail millet husk, sustainable fiber, sound absorption, acoustic panel, composite

## Abstract

The current study deals with the analysis of sound absorption characteristics of foxtail millet husk powder. Noise is one the most persistent pollutants which has to be dealt seriously. Foxtail millet is a small seeded cereal cultivated across the world and its husk is less explored for its utilization in polymer composites. The husk is the outer protective covering of the seed, rich in silica and lingo-cellulose content making it suitable for sound insulation. The acoustic characterization is done for treated foxtail millet husk powder and polypropylene composite panels. The physical parameters like fiber mass content, density, and thickness of the composite panel were varied and their influence over sound absorption was mapped. The influence of porosity, airflow resistance, and tortuosity was also studied. The experimental result shows that 30-mm thick foxtail millet husk powder composite panel with 40% fiber mass content, 320 kg/m^3^ density showed promising sound absorption for sound frequency range above 1000 Hz. We achieved noise reduction coefficient (NRC) value of 0.54. In view to improve the performance of the panel in low-frequency range, we studied the efficiency of incorporating air gap and rigid backing material to the designed panel. We used foxtail millet husk powder panel of density 850 kg/m^3^ as rigid backing material with varying air gap thickness. Thus the composite of 320 kg/m^3^ density, 30-mm thick when provided with 35-mm air gap and backing material improved the composite’s performance in sound frequency range 250 Hz to 1000 Hz. The overall sound absorption performance was improved and the NRC value and average sound absorption coefficient (SAC) were increased to 0.7 and 0.63 respectively comparable with the commercial acoustic panels made out of the synthetic fibers. We have calculated the sound absorption coefficient values using Delany and Bezlay model (D&B model) and Johnson–Champoux–Allard model (JCA model) and compared them with the measured sound absorption values.

## 1. Introduction

Noise is one of the most common pollutants which has been given less attention in the past decades. Consistent exposure to noise causes many health hazards like cardiac diseases, hearing loss, distress [1,2]. Noise pollution is now being considered seriously and building acoustics has gained more attention. The demand for quieter, comfortable environment has led the designers and researchers to develop effective noise-control materials [3,4]. Acoustic panels are fiber-reinforced polymer composites used to absorb undesirable sound. Glass fibers and rock wool fibers are used in manufacturing acoustic panels commercially for their excellent mechanical strength, light weight nature, smaller diameter, and good structural form. The glass fibers and other synthetic fibers are discouraged for their ill effects on the environment. The awareness about environmental pollution has led to the development of biodegradable products. Natural fiber-based composites have already been considered effectively in the fields of automobile, aerospace, and construction industries [5]. The advancement in building acoustics has made opportunities for studying the sound absorption characteristics of porous fibers [6]. The acoustic panels made out of natural fibers are less hazardous to human and easily bio-degradable, than those made out of synthetic fibers [7].

The principle of sustainable development has influenced the designers and researchers to consider environmental friendly bio-degradable fibers as an alternative sound absorbing materials for glass and synthetic fibers. Many natural fibers like rice husk [8,9,10,11,12], coir [13], jute [14], kenaf [15], banana pseudo stem [16], hemp [17], sugarcane bagasse [18] have been studied and their feasibility to use in polymer composites and acoustic panels have been studied. We have done extensive literature study on those research articles to access the properties of the fiber which are given more importance.

Foxtail millet is an age-old food crop domesticated from northern China, 8000 years ago [8]. The cultivation of this crop spreads across the world including arid and semi-arid regions of Africa, America, and Asia [19]. More particularly in developing countries like India, its cultivation is encouraged and increased because of its good yield with minimal agricultural inputs, resistance to biotic and abiotic stresses like drought, salinity, and fungal diseases [19,20]. Across the world the foxtail millet production is 22.0 lakh ton per year and in India the production is 0.5 lakh ton per year. The foxtail millets have 72–75% hulling percentage. Thus husk production is 5.5–6.16 lakh ton per year across the world and 0.125–0.14 lakh ton per year in India.

Foxtail millet husk is the outer protective covering of the grain and it is removed during the milling process. The foxtail millet husk fiber has appreciable bio-chemical and mechanical properties for utilization in bio polymer composites. The cellulose and hemicellulose content of the fiber (untreated) was measured as 46% to 50% and 30% to 34% respectively. The silica content of the husk was measured as per IS 1917 (Part 3): 1992 and found to be 12%, comparable with other natural fibers used for building insulation. The husk of the foxtail millet is less explored for using as a raw material in the manufacturing industries. Also the high lignin and silica contents make the husk not feasible for livestock feed [21,22]. Thus, an attempt has been made to study the sound absorption characteristics of the foxtail millet husk powder composite to utilize the agricultural waste effectively.

When the sound waves incident on the porous composite material, the air molecules at the surface of the composite and pores within the composite structure are forced to vibrate. At the walls of the pores and tunnels within the structure, because of the thermal and viscous losses, part of the energy is converted into heat. Also the fibers in the composite vibrate and rub together with the propagation of sound waves and a part of the energy is lost because of friction. The porosity, airflow resistance and tortuosity are the prime properties influencing the sound absorption. Porosity is the measure of volume of pores in the composite, and it shows how the sound waves penetrate into the composite structure and interact with the air molecules trapped within the pores of the composite. The airflow resistance is the measure of resistance experienced by the air when it passes through the composite. Tortuosity is the measure of shape of the air void passage and it reduces the speed of the sound within the porous composite. This apparently reduces the high-frequency limit by increasing the length of the path of the sound waves propagating through the composite. Density of the composite and fiber mass content directly influences the porosity and airflow resistance [23,24]. The thickness of the sample influences the airflow resistance and tortuosity. To map the influence of the porosity, airflow resistance and tortuosity of the composite in sound absorption characteristics of the composite, the parameters like density, fiber mass content, and thickness of the sample are altered and respective sound absorption coefficient values are measured experimentally using an impedance tube. The models Delany and Bazley and Johnson–Champoux–Allard were used to calculate the sound absorption coefficient values and the results are compared with the experimental values. The parameters considered for the calculation of sound absorption coefficient values were different for Delany and Bazley and Johnson–Champoux–Allard model. 

The mechanism and sound absorption in low-frequency range are not similar to the sound absorption in high-frequency range because the low-frequency sound waves have longer wavelength and are difficult to be absorbed. Necessarily we are bound to find improvement techniques like introducing air gap or perforated plates or hard backing materials or surface coatings for improving the efficiency of acoustic absorption in low-frequency range. In this research we studied the influence of introducing backing material and air gap for the acoustic panel in view to improve its efficiency in low-frequency sound range. Backing materials are hard materials placed as the base of the panel. 

Wide range of natural fibers are studied for utilizing in the acoustic panels but all the fibers have not reached the industry level because of many short-comings with respect to durability. Effective pretreatment of fibers is more important in case of product development from natural bio-fibers [16]. The fiber treatment should increase the interphase adhesion, moisture resistance, and microbial resistance [25]. It is said that alkali treatment reduces the fiber diameter, improves the fiber quality, adhesion property, and microbial resistance. The reduction in fiber diameter improves the tortuous path, airflow resistance, and high surface area. Our focus in this research is to improve the sound absorption in low-frequency range, thus we tried to incorporate number of improvement techniques to the best of the fiber utilization. There are many fiber treatments like mercerization or alkaline treatment, graft copolymerization, and plasma treatment. But alkaline treatment suits best for this research as it improved the fiber quality and was found to be economical. [25] We tried various concentrations of NaOH solution and also duration of the alkali treatment and finalized the one having the minimal deterioration effect on the fiber as the best suited concentration and duration. The sound absorption of the panel for wide sound frequency range makes the acoustic panel more effective. The effective sound absorption of any composite material can be achieved when it has more tortuous path, higher surface area, higher flow resistivity, and low porosity within the optimal range [26].

## 2. Experiments

### 2.1. Materials and Preparation of the Specimens

**Figure 1:**
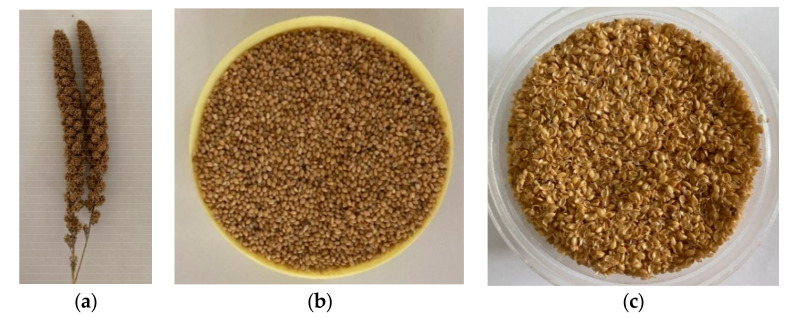
Foxtail millet (**a**) panicle; (**b**) grain; (**c**) husk.

For the experiment, the husks removed from the grains were used. The Figure 1 shows the grains and husk of the foxtail millet. The foxtail millet husks were washed in running water for about 1 h to remove dirt and organic impurities. The washed husk was dried in an oven at a temperature of 100 °C for 1 h. Then the husk is free from water and impurities. We followed the alkali treatment for improving the mechanical properties of the husk. The main objective was to remove the impurities, wax, and reduce the lignin content to a certain extent as these contents will hinder the fiber matrix adhesion. In addition, the fiber size has to be reduced considerably without affecting the strength of the fiber. Sodium hydroxide (NaOH) solution was selected for the treatment of foxtail millet husk. Both the concentration of NaOH solution and duration of treating the husk should be considered for the treatment.

The NaOH concentrations at 0%, 2%, 4%, 6%, 8%, and 10% were tried for treating the husk and the different durations of soaking the husk like 1, 2, 3, 4, and 5 h were tried. The 6% concentration NaOH solution for 4 h soaking duration gave the best result. After 4 h, the fibers were removed and washed with diluted HCl for neutralizing. After that, the fibers were washed in normal running water for 1 h to remove the chemicals completely. Then the fibers were sun dried for 48 h followed by oven drying at a temperature of 80 °C for 1 h. 

**Figure 2:**
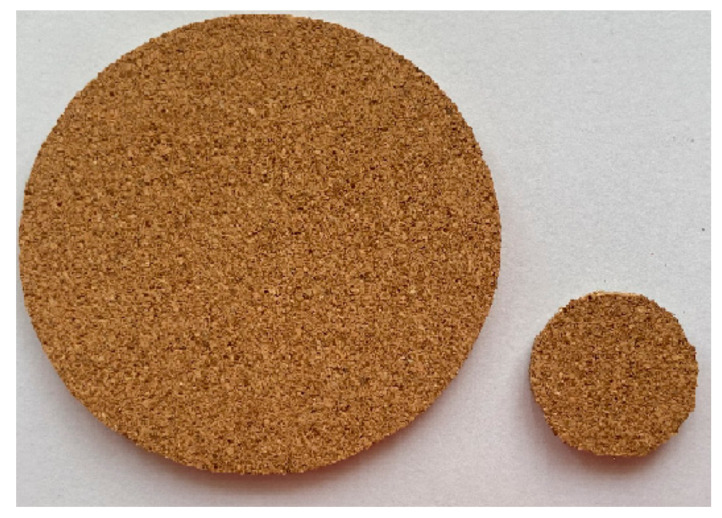
Foxtail millet husk powder composite sample for measuring sound absorption.

The treated husk were pulverized to grain of size 0.6 mm and 0.4 mm. The basic procedure followed in making the acoustic panel was the hot compression method. Polypropylene in powder form with density 898 kg/m^3^, melting point 160 °C, melt flow rate 1.6 g/10 min at 230 °C, tensile modulus 1.3 GPa, and tensile strength 28 MPa was used as the resin. The panel densities tried were 300 kg/m^3^, 320 kg/m^3^, 340 kg/m^3^, 360 kg/m^3^, and 380 kg/m^3^. The fiber content by weight varied from 20% to 60% with the interval of 10%. The polypropylene in powder form was mixed with the fibers manually. The melting point of the resin is 160 °C. In the process of hot compression, with application of temperature 160 °C and pressure, the polypropylene melts and blends with the fiber. The husk powder were mixed with the resin and the mixer was compressed in three stages. Initially, the pressure was maintained as 4.90 MPa for 10 min and 3.92 MPa for 5 min and 2.94 MPa for 5 min. The compressive pressure and duration were decreased gradually and the total time of the application of pressure and temperature was 20 min. The temperature was maintained at 160 °C throughout the process. Thus the panel was made in the size of 300 × 300 mm with varying thicknesses of 15 mm, 20 mm, 25 mm, 30 mm, and 35 mm for samples with varied densities and fiber content. Figure 2 represents the composite panel prepared for measuring sound absorbtion.

### 2.2. Measurement of Sound Absorption Coefficient

**Figure 3:**
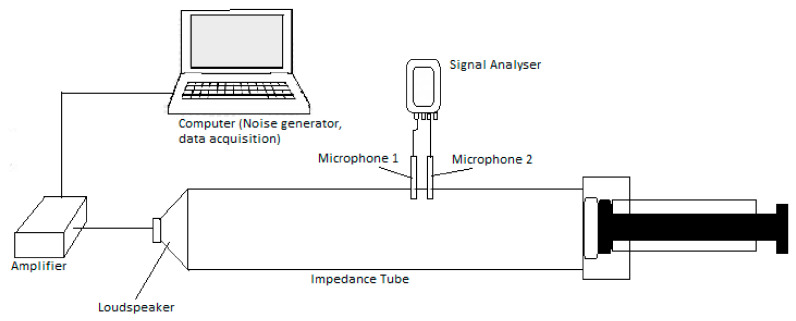
Impedance tube experimental setup.

The sound absorption coefficient values for the prepared samples were measured using impedance tube model Bruel & Kjaer & 2716-c, Nærum, Denmark. Two-microphones transfer matrix method was adopted for sound absorption coefficient measurement. The normal incidence absorption coefficient and normal specific impedance were measured as per ISO 10534-2, ASTM E1050 and ASTM E2611 respectively. The sample to be tested for sound absorption is placed in the receiver end. The sound absorption is based on the measurement of interactions between the acoustic pressures of incident and reflected sound waves [27]. The sound waves in the form of plane waves are generated from the transmitting end through the speaker. Power spectral densities and cross spectral densities are measured at the two microphone locations [27]. The experimental setup is as shown in Figure 3. 

The calculation for sound absorption coefficient is as follows:

The transfer function *H* is the ratio of auto and cross spectral densities.

(1) $$H=\;\frac{S_{12}}{S_{11}}$$

The complex reflection coefficient (*R*) is given by

(2) $$R=\frac{H-e^{-jks}}{e^{-jks}-H}e^{j2k\left(t+s\right)}$$

where, *L*—the distance from the sample face to the first microphone; *S*—the distance between the microphones = $k-\frac{2\pi f}{c}$; *F*—the frequency of the incident sound; *C*—the speed of sound 

The normal incident incidence absorption coefficient can be calculated as follows:

(3) $$\alpha =1-{\left|R\right|}^{2}$$

The normal specific acoustic impedance ratio is given by:

(4) $$\frac{z}{\rho c}=\;\frac{\left(1+R\right)}{\left(1-R\right)}$$

### 2.3. Measurement of Porosity

Porosity is the ratio of volume of air within the sample to the total volume of the sample. The porosity is measured using the Archimedes principle as per BS EN 993-1: 1995 [28]. The dry mass of the sample (*m*_1_) was measured immediately after oven drying the sample at a temperature of 100 °C for 24 h. For the mass of immersed sample (*m*_2_), the sample was completely immersed in distilled water in a suspended cage connected to a hydrostatic weigh balance. The mass of the water-saturated sample (*m*_3_) was measured after removing the immersed sample [29]. The porosity is calculated using Equation (5).

(5) $$\phi =\frac{m_{3}-m_{1}}{m_{3}-m_{2}}\times 100$$

Theoretically the porosity was calculated using Equation (6)

(6) $$\phi =100-\rho_{c}\left(\frac{\gamma }{\rho_{f}}+\frac{g}{\rho_{m}}\right)$$

where: ϕ—porosity (%); γ—mass fraction of foxtail millet husk powder (weight %); *ρ_f_*—specific gravity of the foxtail millet husk powder, and *ρ_m_*—density of the resin

*g*—mass fraction of polypropylene resin (weight %).

### 2.4. Models for Calculating Sound Absorption Coefficients

The sound absorption coefficient values measured experimentally were compared with the values estimated using theoretical models. We have chosen the empirical models proposed by Delany and Bazley [30] and Johnson, Champoux, and Allard [31]. In both the models, the sound absorption coefficients were estimated in terms of characteristic impedance *Z_c_* (*ω*) and the wave number *K* (*ω*). The values of characteristic impedance and the boundary conditions surrounding the porous layer determine the ability of the sound waves to penetrate through the layer. The characteristic wave impedance of the sound wave in the absorbing material is expressed as a function of porosity, airflow resistance, and tortuosity. The characteristic impedance in the frequency-dependent models treats the viscous and thermal effects in the mechanism of sound absorption separately. The difference between the models lies in the parameters they have considered for calculating characteristic impedance and wave number. Also, the assumption of the frame of the sound absorber as rigid or elastic varies with respect to the models.

The viscous frictional losses in the oscillatory flow in the porous medium are pronounced below as Biot frequency [32].

$$\mathrm{Biot}\;\mathrm{frequency}=\frac{8\eta }{S^{2}\rho_{0}}$$

where, *η* is the dynamic viscosity, $\;\rho_{0}$ is the density of air, and *S* is the pore size. Above this Biot frequency, the viscous friction is relatively small and inertial forces become predominant. Biot frequency is the prime factor which separates viscous and inertial flow regime in the pore. The complex reflection coefficient *R* is the ratio of reflected complex pressure to incident complex pressure. These complex pressures have both magnitude and phase component and are dependent on frequency of sound [33]. In theoretical models, the characteristic impedance and wave number are linked to dynamic density and complex compressibility. 

#### 2.4.1. Delany and Bazley Model

The model developed by Delany and Bazley in 1970 [34] is a simple and semi-empirical approach for calculating the surface characteristic impedance and the propagation constant as a function of the air flow resistivity of porous materials. This model expressed the acoustical properties as a function of airflow resistivity alone. This model utilizes the following equations for the calculation of acoustical characteristics [34].

(7) $$Z_{C}\left(\omega \right)=\rho_{O\;}c\left(1+0.0571{\left(X\right)}^{-0.754\;}-j0.087{\left(\frac{\rho_{0}f}{\sigma }\right)}^{-0.732\;}\right)$$

(8) kCω=ωc1+0.0978X−0.7 −j0.189ρ0fσ−0.595 

(9) $$X=\frac{\rho_{0}f}{\sigma }$$

(10) $$Z_{s}=-jZ_{c}\cot\left(kd\right)$$

(11) $$R=\frac{Z_{s}-\rho_{0}c_{0}}{Z_{s}+\rho_{0}c_{0}}$$

(12) $$Sound\;absorption\;coefficient,\;\alpha =1-{\left|R\right|}^{2}$$

where, *ρ*_0_ is the density of air; *c*_0_ is the speed of sound in the air; *Z_c_* (*ω*) is the characteristic impedance; *K_c_* (ω) is the propagation constant; *f* is the frequency; ω is the angular frequency; *σ* is the static airflow resistivity; *R* is sound pressure reflection coefficient; *Zs* is the surface impedance; *d* is the thickness of the sample.

#### 2.4.2. Johnson–Champoux–Allard (JCA) Model

Allard and Champoux [31] in 1992 proposed a phenomenological model for propagation of sound in porous materials. For the estimation of acoustic absorption coefficient, this model incorporates the parameter air flow resistivity (*σ*), porosity (φ), tortuosity (α_∞_), viscous characteristic length (Λ), and thermal characteristic length (Λ′). This is a rigid frame model, assuming the frame is rigid and heavier than the fluid in the porous medium. This model defines equivalent bulk and density module as follows:

(13) $$\rho \left(\omega \right)=\;\alpha {\;}_{\infty }\rho_{0}\left[1+\frac{\sigma \emptyset }{j\omega \rho_{o}\alpha_{\infty }}{\left(1+\frac{4i\alpha_{\infty }^{2}\eta \omega \rho_{0}}{{\left(\sigma \Lambda \emptyset \right)}^{2}}\right)}^{1/2}\right]$$

(14) $$K\left(\omega \right)=kp_{0}{\left(k-\left(k-1\right){\left[1+\frac{8\eta \alpha_{\infty }\emptyset }{\Lambda^{\prime }{}^{2}\emptyset i\omega \rho_{0}\alpha_{\infty }N_{pr}}{\left(1+\frac{4i\alpha_{\infty }^{2}\eta N_{pr}\omega \rho_{0}}{{\left(\sigma^{\prime }\Lambda^{\prime }\emptyset \right)}^{2}}\right)}^{\frac{1}{2}}\right]}^{-1}\right)}^{-1}$$

where *ρ*_0_ indicates the air density (kg/m^3^), *N_pr_* is the Prandtl number (0.71), *η* is the viscosity of the air (1.85 × 10^−5^), *k* is the ratio of the specific heat capacity (1.4), and *ω* is the angular velocity (1/s).

(15) $$Z_{c}\left(\omega \right)=\frac{1}{\emptyset }\sqrt{\rho_{\left(\omega \right)}.K_{\left(\omega \right)}}$$

(16) $$K_{\left(\omega \right)}=\;\omega \sqrt{\frac{\rho_{\left(\omega \right)}}{K_{\left(\omega \right)}}}$$

(17) $$Z=\;Z_{c}\left(\omega \right).\cot\left(K_{c}\left(\omega \right)xd\right)$$

(18) $$R=\frac{Z_{s}-\rho_{0}c_{0}}{Z_{s}+\rho_{0}c_{0}}$$

where, *R* is the sound pressure reflection coefficient, *Z_s_* is the surface impedance, *d* is the thickness of the sample.

The sound absorption coefficient α is given by,

(19) $$\alpha =1-{\left|R\right|}^{2}$$

## 3. Results and Discussion

Sound absorption coefficient was measured for various samples using impedance tube method varying the factors like density, fiber mass content, and thickness. The incorporation of granular fiber in acoustic panel increases the flow resistivity to have good absorption in low-frequency sound range (200 Hz to 1000 Hz). The airflow resistance and tortuosity are vital factors considered for designing acoustic panels. The parameters so far identified to have remarkable influence over these factors are fiber diameter or grain size, porosity, bulk density, fiber content, thickness of the layer, and air gap. With respect to the natural bio-fibers, the parameters like fiber diameter, bulk density, sample thickness, fiber content by weight percentage differ much between each type of bio-fiber. An attempt has been made to map the variation of sound absorption coefficient with physical parameters like bulk density, fiber mass ratio, and thickness of the composite. Porosity of the composites was also calculated for all samples so as to study how the variation occurs in the prime physical parameters like airflow resistance and tortuosity.

### 3.1. Chemical Properties of Foxtail Millet Husk

The chemical properties like silica content, cellulose, hemi-cellulose, and lignin contents of the selected fibers were measured by the method described by Bledzki et al. 2008 [35]. From the literature study, we found that the chemical properties influence the strength and durability of the fiber [35]. The treatment of the husk removed considerable amount of lignin and hemi-cellulose content. The aim of treating the fiber is to reduce the diameter of the fiber effectively, simultaneously increasing the strength of the fiber. The chemical parameters measured before and after the chemical treatment are given in Table 1. From the literature study, it is assumed that increase in cellulose content improves the strength of the fiber. Treating the husk with 6% NaOH solution for 4 h improved the bonding of the fiber with the resin which we observed practically while making the composite panel. 

### 3.2. Scanning Electron Microscpe (SEM) Analysis of the Foxtail Millet Husk Powder Composites

**Figure 4:**
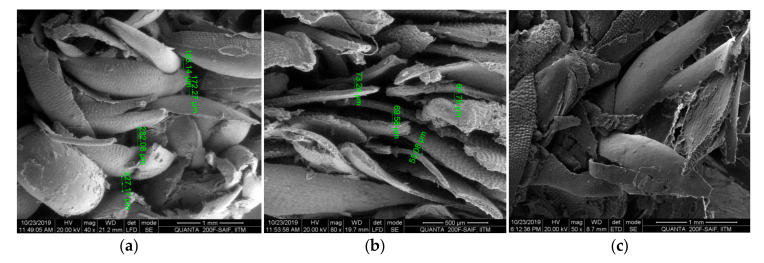
The SEM images of foxtail millet husk powder composites. (**a**) Composite with density 320 kg/m^3^ (**b**) Composite with density 850 kg/m^3^. (**c**) Surface of the composite.

SEM images of the foxtail millet husk powder composites are shown in Figure 4. The Figure 4a shows the husk powder arrangement in the composite with the density 320 kg/m^3^ and Figure 4b shows the composite with 850 kg/m^3^. From the image it can be noted that there were patches of resin layers in the husk powder surface which showed that the resin was not coated uniformly. The high lignin content in the husk powder hindered the cohesion between the husk powder and the binder. Thus, it has to be taken care in future studies. Also we noticed mild strain in the husk powder structure which might be due to the degree of temperature in the hot compression process. We noticed more strain in the fiber in the vicinity of the composite surface (Figure 4c). The temperature and duration of hot compression also influenced the sound absorption which are to be dealt in detail in future studies. 

### 3.3. Mass Fraction of Foxtail Millet Husk Powder and Sound Absorption

**Figure 5:**
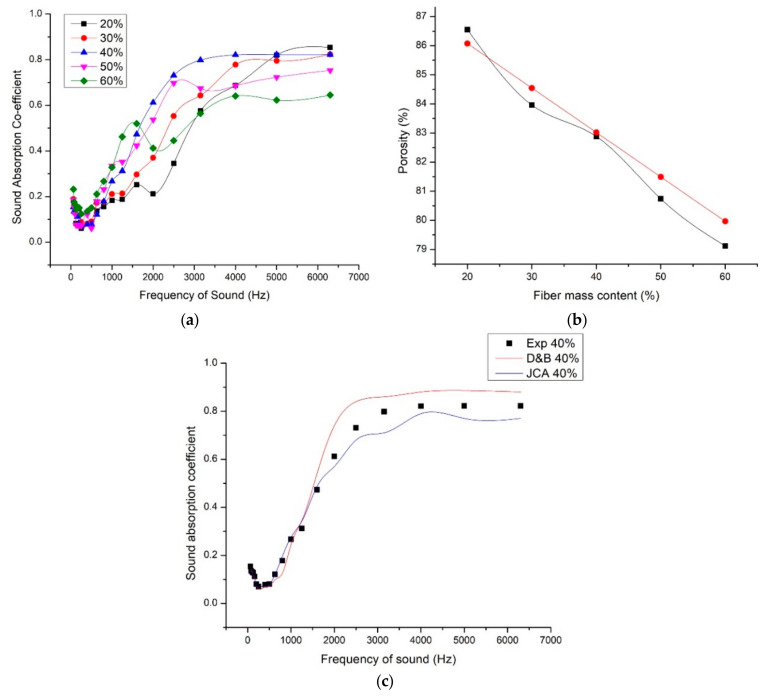
(**a**) Variation of sound absorption coefficient with respect to fiber mass content. (**b**) Change in porosity with respect to fiber mass content [black—measured, red—calculated]. (**c**) Comparison of experimental SAC values with the estimated SAC values using Delany and Bezley model (D&B) and Johnson–Champoux–Allard model (JCA).

Composite with fiber mass fraction of 20%, 30%, 40%, 50%, and 60% were prepared under necessary technical conditions that consisted of bulk density 300 kg/m^3^. The thickness of the samples was maintained as 15 mm. The porosity, fiber content, and bulk density are inter-related and influenced each other. With the increase in fiber content in terms of fiber mass fraction in composite with constant dimension, number of fibers per unit volume increases, reducing the pore size and pore volume in the composite. The graph (Figure 5b) shows the comparison of the measured and calculated porosity values. Experimentally the porosity was measured using Archimedes Principle (Equation (5)) and theoretically Equation (6) is used to calculate the porosity of the samples. When the fiber mass content of the foxtail millet husk powder is increased from 20% to 60%, the porosity decreases from 86% to 80%. As discussed earlier, the sound absorption occurs because of thermal and viscous losses of the air molecule collusions within the pores. Mean free path is the average distance travelled by the air molecule before colliding with each other. These collisions pass the energy through the porous medium. From the graph (Figure 5a), it could be seen that 30% fiber mass content gave maximum sound absorption coefficient for the frequency range 4000 Hz to 6000 Hz. But for grading the composite to highly efficient sound absorber, the sound absorption coefficient should be appreciably high for a wide range of frequencies (i.e., even in low-frequency range 200 Hz to 1000 Hz, the sound absorption coefficient was appreciably high). Considering that, 40% fiber mass fraction composite showed good sound absorption coefficient for wide range of sound frequencies. When the fiber mass content is increased beyond 40%, the pore sizes are reduced further and sound waves could not pass through the medium and interact with the trapped air molecules in the pores. Also it is visible in Figure 5a that the sound absorption coefficient increases with increase in frequency independent of the difference in the fiber mass fraction. This shows that sound absorption at high frequency of sound is easier than at low frequency of sound. This makes necessary to understand the mechanism of sound absorption. Low frequency sound has larger wavelength compared to high-frequency sound, making it more difficult to absorb. For the selected foxtail millet husk fiber 40% fiber mass fraction was found to be efficient in sound absorption behavior. Further increase in the fiber content reduced the pores formed in the composite structure and reduced the sound absorption. In the case of less fiber mass fraction, pore formation was discontinuous and proper inter-connections between the pore and outer surface were not formed resulting in low sound absorption. The optimum quantity of pores with optimum size resulted in proper vibration of air molecules between the pores in the incident sound waves and thus frictional loss between the fiber (pore walls) and enclosed air particles transformed the sound energy. The resin content and nature is equally important because it decides the bonding of fibers thus confirming the continuation of pores which is vital in sound absorption. Fixing optimal fiber fraction confirms required quantity of pores in the form of cavities, channel paths or interstices, effective pore size, interconnection of pores for proper propagation of sound [36].

For analyzing the overall behavior of the composite in sound absorption for wide sound frequency range, the average sound absorption value and noise reduction coefficient (NRC) values were calculated and compared for all fiber mass fractions. From the Table 2 it could be noted that 40% fiber mass fraction of foxtail millet husk powder gave an efficient sound absorption with average sound absorption coefficient value of 0.33 and NRC 0.257.

We have used the D&B model and JCA model to calculate the sound absorption coefficient values of the composite of density 300 kg/m^3^, fiber mass content 40%, and thickness 15 mm. We have selected these parameters because these values gave maximum NRC values and average sound absorption coefficient values. D&B model predicted SAC values closer to experimental values until 1650 Hz. The porosity was close to unity for 40% fiber mass content thus the simple D&B model predicted closer values. D&B model expresses the characteristic impedance and wave number in terms of airflow resistivity. Whereas JCA model expresses the characteristic impedance and wave number as a function of effective bulk density and bulk modulus. As the porosity was close to unity, it was assumed that л′ = 2л [37]. The JCA predicted more close SAC values compared to D&B model. The peak SAC value estimated was also close to the measured value.

### 3.4. Influence of Apparent Density on Sound Absorption

**Figure 6:**
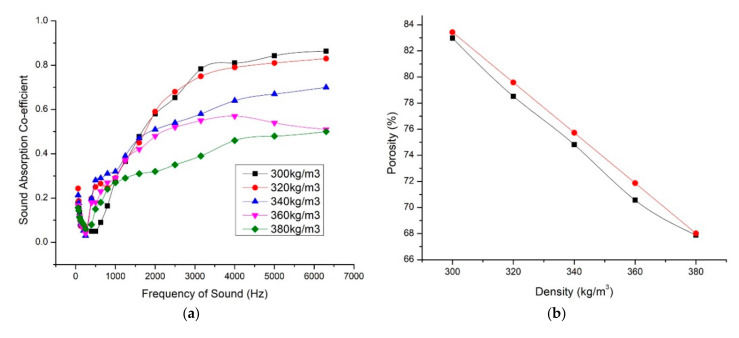

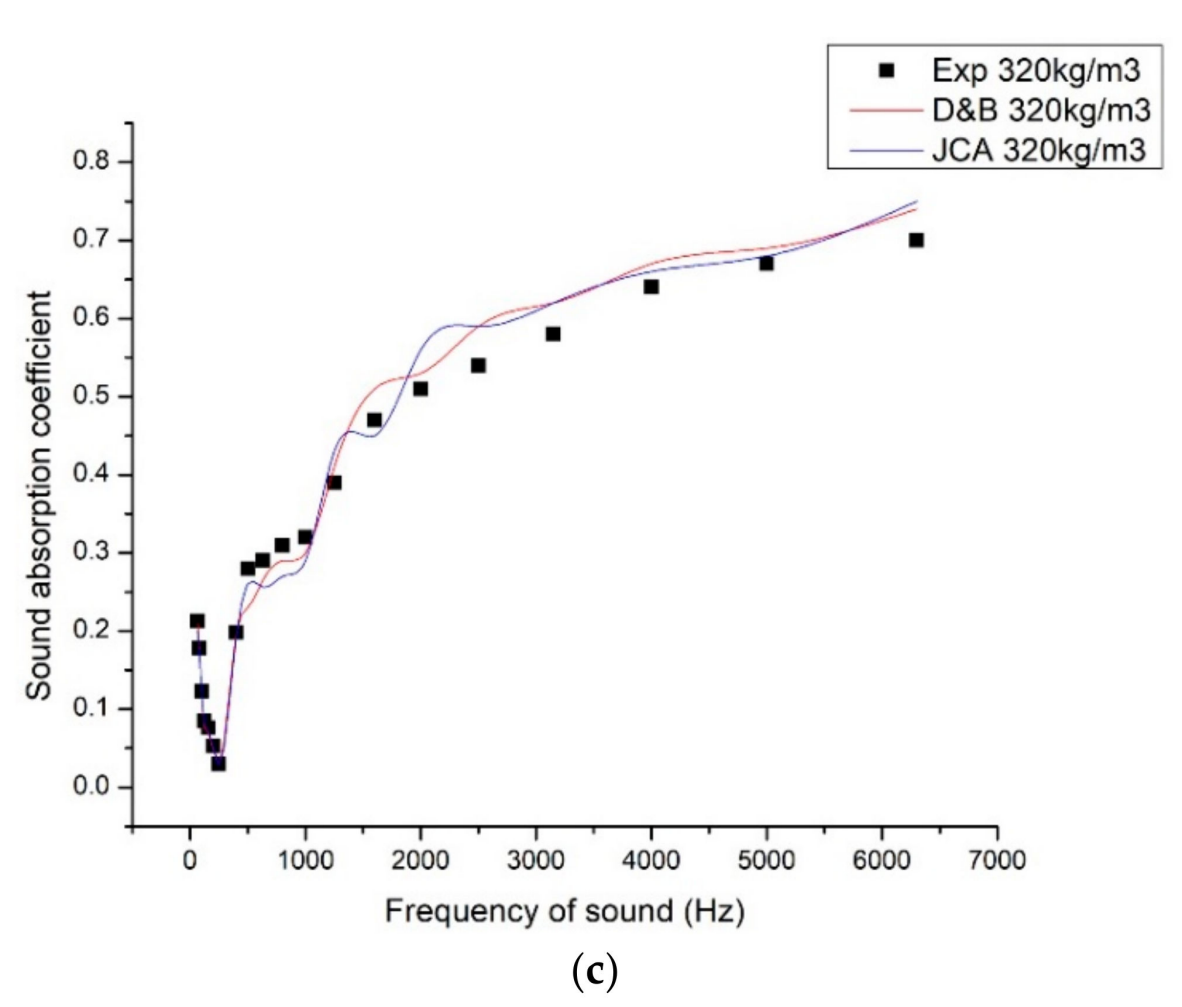
(**a**) Variation of sound absorption coefficient with respect to volume density. (**b**) Change in porosity with respect to volume density [black—measured, red—calculated]. (**c**) Comparison of experimental SAC values with the estimated SAC values using Delany and Bezley model (D&B) and Johnson–Champoux–Allard model (JCA).

Under technological conditions, the densities of the composite varied as 300 kg/m^3^, 320 kg/m^3^, 340 kg/m^3^, 360 kg/m^3^, and 380 kg/m^3^. For all the densities, the fiber mass fraction was fixed as 40% and material thickness 15 mm. From the Figure 6b it could be seen that change in density varied the porosity drastically. When the density increased from 300 kg/m^3^ to 380 kg/m^3^, the porosity decreased from 83% to 68%. With increasing the density of the composite, the fibers are more closely packed. When the fibers come closer, the pore size and volume reduced.

From the graph (Figure 6a) we studied that the lowest dense composite (300 kg/m^3^) gave the highest sound absorption coefficient 0.86 for 6500 Hz. But in low-frequency ranges, which is for sound frequency range below 1000 Hz, the 300 kg/m^3^ composite showed less sound absorption coefficient values. But the high-density composite behaved well in low-frequency ranges and the sound absorption coefficient values decreased with increase in sound frequency. For effective sound absorption in low-frequency range, the airflow resistance is equally important as the porosity. The absorption of sound energy in the porous material depends on the damping mechanism in the solid and frictional viscous losses between air molecules and fiber walls. The performance of the acoustic absorbance is improved when the transfer of energy from air molecules to the structure is enhanced. When the fibers are closely packed, the airflow resistivity is also increased. The airflow resistance is one of the properties that determines both the sound absorbing and sound transmitting properties of a material. The resistance offered by the fiber for the inflow sound waves reduces its speed and thus supports sound absorption coefficient. This was clearly observed in the selected foxtail millet husk powder composite. But the height of the curve also reduced for high-density composites. Table 3 gives the average sound absorption coefficient and NRC of the samples. It is clear that 320 kg/m^3^ sample gives best sound absorption coefficient for varied sound frequency range. The curve is relatively wide which shows overall best sound absorption result. The SAC values for 300 kg/m^3^ and 320 kg/m^3^ were close to each other in high-frequency range. The 300 kg/m^3^ panel gave low SAC values in low-frequency range. When the density of the fiber was further increased, the performance was reduced because of reduced porosity values thus the reflection of sound waves was more than penetration and interaction with the matrix. 

We have used the D&B model and JCA model to calculate the sound absorption coefficient values of the composite of density 320 kg/m^3^, fiber mass content 40%, and thickness 15 mm. We have selected these parameters because; these values gave maximum NRC and average SAC values. As discussed earlier, the parameters considered for estimating the SAC values were different. The peak SAC in 1650 Hz was predicted more closely to the experimental value by JCA mode. In higher frequency ranges, D&B model predicted higher values.

### 3.5. Influence of Sample Thickness on Sound Absorption

**Figure 7:**
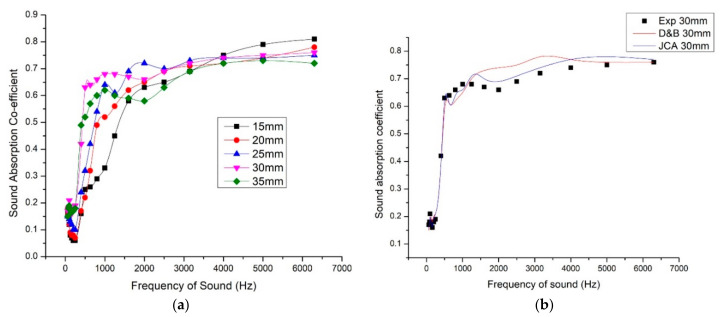
(**a**) Variation of sound absorption coefficient with respect to thickness. (**b**) Comparison of experimental SAC values with the estimated SAC values using Delany and Bezley model (D&B) and Johnson–Champoux–Allard model (JCA).

Sample thicknesses 15 mm, 20 mm, 25 mm, 30 mm, and 35 mm were taken into consideration, fixing the density 320 kg/m^3^ and 40% fiber mass content as constants. This clearly mapped the role of thickness in sound absorption behavior. With the increase in thickness, the peak sound absorption coefficient moved toward low-frequency range. Particularly, the peak value increased for frequency range 500 Hz to 1000 Hz. Though the 35-mm sample gave better sound absorption in low-frequency range, the curve was wide for 30-mm sample which proved to have the better sound absorption for wide range of sound absorption. As we discussed earlier, the high-frequency sound has shorter wavelength which is absorbed in the vicinity of the surface of the panel and low-frequency sound has longer wavelength which has to travel a longer distance for absorbance. From the graph (Figure 7), it can be observed that with the increase in thickness, the deviation in sound absorption coefficient is more in the frequency range 500 Hz to 2000 Hz. For the frequency range 3000 Hz to 6500 Hz, the sound absorption coefficient does not differ much with increase in sample thickness. Airflow resistance also depends on the sample thickness. 

With the average sound absorption coefficient and NRC values calculated, the 35-mm thick sample gave better results. Table 4 gives clarity for the overall performance of the samples with thicknesses 15 mm, 20 mm, 25 mm, 30 mm, and 35 mm. The thickness and density have considerable influence over airflow resistance and thus on sound absorption. Higher the thickness and density, the more effective was the sound absorption coefficient in the low and mid frequency ranges [38,39].

We have used the D&B model and JCA model to calculate the sound absorption coefficient values of the composite of density 320 kg/m^3^, fiber mass content 40%, and thickness 30 mm. We have selected these parameters because these values gave maximum NRC and average SAC values. Here JCA model predicted more close values because it considered more non-acoustic parameters for the calculation of characteristic impedance and wave number.

### 3.6. Influence of Air Gap and Backing Material 

**Figure 8:**
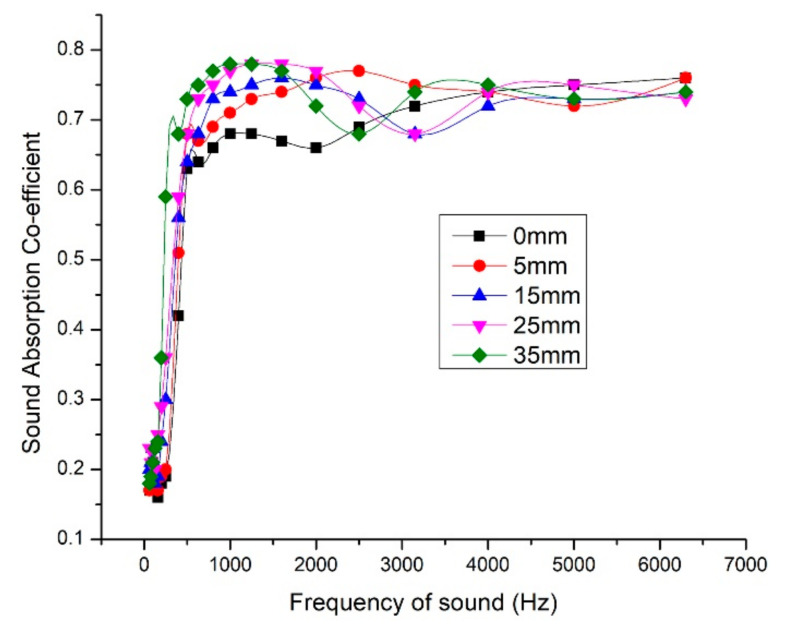
Variation of sound absorption coefficient with varying air gap thickness.

From Figure 6a, increase in density until certain limit, moved the peak sound absorption coefficient toward mid and low-frequency ranges. But the least dense composite (300 kg/m^3^) gave the highest peak sound absorption coefficient value of 0.86 for 6500 Hz sound frequency. At the same time, the least dense composite was found to be less efficient in low-frequency range. Composites with densities 340 kg/m^3^ and 360 kg/m^3^ were found to be more efficient compared to lower density composites with frequency range till 1500 Hz. With these observations, to make the composite more efficient for both higher and lower frequency ranges, we decided to combine the low-density composite and high-density composites with air-gap in between. The panel was made in such a way that the 320 kg/m^3^ composite faces the sound incidence and 850 kg/m^3^ composite as backing material. The maximum NRC value of 0.54 was observed for composite with fiber mass content 40%, density 320 kg/m^3^, and thickness 30 mm. We tried giving 5-mm thick composite with density 850 kg/m^3^ as backing material and varying air gap thickness. For air gap thickness of 35 mm, we achieved an NRC value of 0.70 and average sound absorption coefficient value of 0.63. Figure 8 shows the SAC variation with respect to air gap thickness variation.

As discussed earlier, the pore size and airflow resistance are inter-related and have massive influence over sound absorption. The low-frequency sound waves have longer wavelength and tedious to absorb. The longer wavelength makes the low-frequency sound to travel longer distance than high-frequency sound waves. So leaving an air gap and the rigid backing material eventually moved the peak sound absorption coefficient toward the low-frequency end and overall efficiency was also improved. The air gap thickness varied as 0 mm, 5 mm, 15 mm, 25 mm, and 35 mm and the improvement in the sound absorption is studied. As the air gap increased, the peak sound absorption coefficient moved toward the low-frequency range. For 35-mm air gap and 5-mm backing material, we could achieve sound absorption coefficient of 0.73 for sound frequency 500 Hz. As we desired, the performance of the panel in low-frequency range improved and is comparable with the commercially available acoustic panels. Table 5 give the details of NRC and average SAC values for various air gap thickness and Table 6 gives the NRC values for prevailing fibers used for sound insulation.

We have tabulated the NRC values of various natural fibers and glass wool panels. The NRC value measured for the FMH panel of 320 kg/m^3^, 40% fiber mass content, and 30-mm thickness was 0.54. When 35-mm airgap and backing material was introduced, we could achieve NRC value of 0.7. Further study has to be studied in the resin matrix and hybridization with other fibers for improving the sound absorption efficiency.

## 4. Conclusions

The experimental result shows that the porosity and airflow resistance have major influence in sound absorption of the composite. The fiber mass content and density of the composite when increased, altered the porosity, pore size, and fiber arrangement. In addition, the airflow resistance increased with decrease in porosity. Depending on the nature of the fiber, increase in fiber mass content and density improves the sound absorption coefficient until certain limit. When the density is increased beyond this critical limit, the sound wave could not pass through the structure and reflected back, hence affecting the performance.

As far as the foxtail millet husk powder is concerned, when the fiber mass content was increased above 40%, the peak sound absorption coefficient values start decreasing. The peak sound absorption coefficient value was attained for sound frequencies above 2500 Hz. 

For the composite panel with 40% fiber mass content which we found optimum for foxtail millet husk powder and 15 mm thickness, we increased the bulk density. When the density was increased above 320 kg/m^3^, the overall performance seems to decline. Increase in density increased the airflow resistance which slightly improved the performance in low-frequency sound range.

With the variation in the thickness of the sample, the combined effect of airflow resistance and tortuosity can be analyzed. Increasing the thickness, moved the peak sound absorption coefficient toward low-frequency range. The 30-mm thick sample with 320 kg/m^3^ density and 40% fiber mass content gave appreciable sound absorption. Further increase in thickness reduced the performance and also we could not achieve homogenous structure because of the fiber nature.

In view to improve the efficiency in low-frequency range that is 250 to 500 Hz, we incorporated air gap and rigid backing material. The variation in air gap thickness drastically improved the sound absorption below 500 Hz as we expected. We used foxtail millet husk powder particleboard with density 850 kg/m^3^ as backing material to study the efficiency of pure foxtail millet husk powder without hybridization with other material fibers. By giving 35-mm thick air gap and 5-mm thick rigid backing material we could achieve NRC value of 0.70 and average sound absorption coefficient value of 0.63. This is comparable with the commercially available acoustic panels at present. 

We calculated the SAC values using D&B and JCA models and compared the values with the measured SAC values. Depending upon the frequency of sound, the mechanism of sound absorption may differ. Also the parameters of the composite influencing the sound absorption depend greatly on the frequency of sound. The mechanism of propagation of sound waves in the porous material differs as the frequency of sound differs because the wavelengths of the sound waves are different for different sound frequency ranges. Also the composite made out of the bio-fiber composite was not homogenous and isotropic. So, the calculated SAC values were not closer to the measured values for all sound frequency range.

Further studies are planned to study the mechanical characteristics of the panel, life-time assessment of the panel, and for durability analysis. Also the resin matrix was not uniformly distributed and the bonding should be taken care of and improved. The temperature and duration of heat and compression applied need to be studied in detail to avoid the fiber damage. 

## Figures and Tables

**Table 1:** Bio-chemical components of untreated (6% concentrated NaOH treated for 4 h) and treated foxtail millet husk.

Bio-Chemical Components (%)	Foxtail Millet Husk		
	Untreated	Treated	% Increase/Decrease
Cellulose	48	52	(+) 4
Hemi-Cellulose	33	21	(−) 12
Lignin	11	9	(−) 2

**Table 2:** Noise reduction coefficient (NRC) and average sound absorption coefficient for varied fiber mass content samples (15 mm thickness and 300 kg/m^3^ density).

Fiber Mass Content (%)	125 Hz	250 Hz	500 Hz	1000 Hz	2000 Hz	4000 Hz	NRC	Average Sound Absorption Coefficient
20	0.08	0.06	0.08	0.18	0.21	0.69	0.132	0.217
30	0.16	0.08	0.09	0.21	0.37	0.78	0.187	0.282
40	0.13	0.07	0.08	0.27	0.61	0.82	0.257	0.330
50	0.07	0.07	0.06	0.33	0.53	0.69	0.247	0.292
60	0.14	0.12	0.15	0.32	0.41	0.64	0.250	0.297

**Table 3:** Noise reduction coefficient (NRC) and average sound absorption coefficient for varied volume density samples.

Volume Density (kg/m^3^)	125 Hz	250 Hz	500 Hz	1000 Hz	2000 Hz	4000 Hz	NRC	Average Sound Absorption Coefficient
300	0.09	0.06	0.05	0.28	0.58	0.81	0.243	0.313
320	0.07	0.05	0.25	0.29	0.59	0.79	0.295	0.341
340	0.08	0.03	0.28	0.32	0.51	0.64	0.285	0.310
360	0.09	0.04	0.18	0.29	0.48	0.57	0.247	0.274
380	0.1	0.06	0.15	0.27	0.32	0.46	0.200	0.227

**Table 4:** Noise reduction coefficient (NRC) and average sound absorption coefficient for varied thickness samples.

Thickness (mm)	125 Hz	250 Hz	500 Hz	1000 Hz	2000 Hz	4000 Hz	NRC	Average Sound Absorption Coefficient
15	0.08	0.06	0.25	0.33	0.63	0.75	0.318	0.350
20	0.09	0.07	0.22	0.52	0.65	0.72	0.365	0.378
25	0.13	0.1	0.32	0.64	0.72	0.40	0.445	0.385
30	0.17	0.19	0.63	0.68	0.66	0.74	0.540	0.512
35	0.16	0.18	0.52	0.62	0.58	0.72	0.475	0.463

**Table 5:** Noise reduction coefficient (NRC) and average sound absorption coefficient for varied air gap thickness.

Thickness (mm)	125 Hz	250 Hz	500 Hz	1000 Hz	2000 Hz	4000 Hz	NRC	Average Sound Absorption Coefficient
0	0.17	0.19	0.63	0.68	0.66	0.74	0.54	0.51
5	0.18	0.20	0.68	0.71	0.76	0.74	0.58	0.54
15	0.18	0.30	0.64	0.74	0.75	0.72	0.60	0.55
25	0.20	0.36	0.68	0.77	0.77	0.74	0.64	0.58
35	0.23	0.59	0.73	0.78	0.72	0.75	0.70	0.63

**Table 6:** Noise reduction coefficient (NRC) values of other natural fibers, glass wool and FMH.

Fibers	NRC
Kenaf (60 mm thickness)	0.7
Kenaf (40 mm thickness)	0.55
Wood fiber (60 mm thickness)	0.6
Hemp (30 mm thickness)	0.4
Coir (50 mm thickness)	0.5
Rice husk (25 mm thickness)	0.65
Glass wool panel (25 mm thick)	0.95
Foxtail Millet Husk powder (30 mm thickness)	0.54
